# Uncovering Clinical Risk Factors and Predicting Severe COVID-19 Cases Using UK Biobank Data: Machine Learning Approach

**DOI:** 10.2196/29544

**Published:** 2021-09-30

**Authors:** Kenneth Chi-Yin Wong, Yong Xiang, Liangying Yin, Hon-Cheong So

**Affiliations:** 1 School of Biomedical Sciences, The Chinese University of Hong Kong Hong Kong China; 2 KIZ-CUHK Joint Laboratory of Bioresources and Molecular Research of Common Diseases, Kunming Institute of Zoology and The Chinese University of Hong Kong Kunming China; 3 CUHK Shenzhen Research Institute Shenzhen China; 4 Department of Psychiatry, The Chinese University of Hong Kong Hong Kong China; 5 Margaret K.L. Cheung Research Centre for Management of Parkinsonism, The Chinese University of Hong Kong Hong Kong China; 6 Brain and Mind Institute, The Chinese University of Hong Kong Hong Kong China; 7 Hong Kong Branch of the Chinese Academy of Sciences Center for Excellence in Animal Evolution and Genetics, The Chinese University of Hong Kong Hong Kong China

**Keywords:** prediction, COVID-19, risk factors, machine learning, pandemic, biobank, public health, prediction models, medical informatics

## Abstract

**Background:**

COVID-19 is a major public health concern. Given the extent of the pandemic, it is urgent to identify risk factors associated with disease severity. More accurate prediction of those at risk of developing severe infections is of high clinical importance.

**Objective:**

Based on the UK Biobank (UKBB), we aimed to build machine learning models to predict the risk of developing severe or fatal infections, and uncover major risk factors involved.

**Methods:**

We first restricted the analysis to infected individuals (n=7846), then performed analysis at a population level, considering those with no known infection as controls (ncontrols=465,728). Hospitalization was used as a proxy for severity. A total of 97 clinical variables (collected prior to the COVID-19 outbreak) covering demographic variables, comorbidities, blood measurements (eg, hematological/liver/renal function/metabolic parameters), anthropometric measures, and other risk factors (eg, smoking/drinking) were included as predictors. We also constructed a simplified (lite) prediction model using 27 covariates that can be more easily obtained (demographic and comorbidity data). XGboost (gradient-boosted trees) was used for prediction and predictive performance was assessed by cross-validation. Variable importance was quantified by Shapley values (ShapVal), permutation importance (PermImp), and accuracy gain. Shapley dependency and interaction plots were used to evaluate the pattern of relationships between risk factors and outcomes.

**Results:**

A total of 2386 severe and 477 fatal cases were identified. For analyses within infected individuals (n=7846), our prediction model achieved area under the receiving-operating characteristic curve (AUC–ROC) of 0.723 (95% CI 0.711-0.736) and 0.814 (95% CI 0.791-0.838) for severe and fatal infections, respectively. The top 5 contributing factors (sorted by ShapVal) for severity were age, number of drugs taken (cnt_tx), cystatin C (reflecting renal function), waist-to-hip ratio (WHR), and Townsend deprivation index (TDI). For mortality, the top features were age, testosterone, cnt_tx, waist circumference (WC), and red cell distribution width. For analyses involving the whole UKBB population, AUCs for severity and fatality were 0.696 (95% CI 0.684-0.708) and 0.825 (95% CI 0.802-0.848), respectively. The same top 5 risk factors were identified for both outcomes, namely, age, cnt_tx, WC, WHR, and TDI. Apart from the above, age, cystatin C, TDI, and cnt_tx were among the top 10 across all 4 analyses. Other diseases top ranked by ShapVal or PermImp were type 2 diabetes mellitus (T2DM), coronary artery disease, atrial fibrillation, and dementia, among others. For the “lite” models, predictive performances were broadly similar, with estimated AUCs of 0.716, 0.818, 0.696, and 0.830, respectively. The top ranked variables were similar to above, including age, cnt_tx, WC, sex (male), and T2DM.

**Conclusions:**

We identified numerous baseline clinical risk factors for severe/fatal infection by XGboost. For example, age, central obesity, impaired renal function, multiple comorbidities, and cardiometabolic abnormalities may predispose to poorer outcomes. The prediction models may be useful at a population level to identify those susceptible to developing severe/fatal infections, facilitating targeted prevention strategies. A risk-prediction tool is also available online. Further replications in independent cohorts are required to verify our findings.

## Introduction

COVID-19 has resulted in a pandemic affecting more than a hundred countries worldwide [1-3]. More than 177 million confirmed cases and 3.8 million fatalities have been reported worldwide as of June 19, 2021 [4], while a large number of mild or asymptomatic cases may remain undetected. Given the extent of the pandemic, it is urgent to identify risk factors that may be associated with severe disease, and to gain deeper understanding into its pathophysiology. Accurate prediction of those at risk of developing severe diseases is also clinically important.

Machine learning (ML) approaches are powerful tools to predict disease outcomes and have been increasingly applied in biomedical research. In this study we employed boosted trees (with XGboost) to predict disease outcomes and identify risk factors. This ML approach can capture complex and nonlinear interactions between variables, hence leading to better predictive power in many circumstances. In view of the COVID-19 pandemic, many ML models have been developed for diagnostic or prognostic purposes. For instance, Bayat et al [5] developed a prediction model for COVID-19 infection based on 75,991 veteran patients who were tested for the virus. The prediction was based on boosted trees and predictors included vital signs, hematology measurements, and blood biochemistries. Knight et al [6] built a model to predict in-hospital mortality for patients hospitalized with COVID-19, based on demographics, comorbidities, vital signs, and blood test results. A variety of methods including XGboost, generalized additive model, and LASSO were employed. Chung et al [7] employed deep neural networks to predict the severity of COVID-19 infection based on basic patient information, comorbidities, vital signs, clinical symptoms, and complete blood count. Wynants et al [8] performed a systematic review of COVID-19–related prediction models up to July 1, 2020, covering 169 studies describing 232 prediction models. Several recent reviews have also summarized the applications of ML methods in the study of COVID-19 (eg, [8-11]).

Here we made use of the UK Biobank (UKBB) data to build ML models to predict severity and fatality from COVID-19, and evaluated the contributing risk factors. We built prediction models not only for patients infected but also at a general population level. While predictive performance is the main concern in most previous studies, we argue that ML models can also provide important insights into individual contributing factors and the pattern of complex relationships between risk factors and the outcome. While many have studied risk factors of COVID-19 susceptibility or severity in the UKBB [12-14] or other cohorts (eg, [8,15-18]), most relied on conventional linear models. As such, nonlinear effects and interactions between variables may be missed.

We note that in the UKBB clinical data were collected years before the outbreak of infection in 2020, which may be a limitation. Ideally, the predictors should be measured at the time when the model is intended to be applied (eg, at admission). However, we believe that building ML models with previously collected clinical data is useful for reasons detailed below. First, using previously collected clinical features may facilitate the identification of potential causal risk factors. As the predictors are collected prior to the outbreak, there is no concern about reverse causality. In practice, infection itself will lead to changes in many clinical parameters (eg, glucose, inflammatory markers, liver/renal functions); hence, it is often difficult to tell the direction of effect in cross-sectional studies. We hypothesize that this study will identify general or “baseline” risk factors or laboratory measurements that may be (causally) predictive of outcome. Second, the UKBB is a huge population-based sample (N=~500,000), and the rich clinical data collected previously enable ML models to be developed at the general population level. Importantly, there is a relative lack of such population-level ML prediction models to identify who may be at risk of developing severe COVID-19 infections. We hope this study will fill the gap, as this may have implications for prioritizing individuals for specific prevention strategies (eg, vaccination) and diagnostic testing under limited resources.

In this study we performed 4 sets of analysis. In the first 2 sets, we built ML models to predict the severity and mortality of COVID-19 among those who are tested positive for the virus. In this setting, predictive performance is of secondary concern (as predictors were not assessed at or during admission), but the predictive performance can shed light on to what extent *baseline* (prediagnostic) clinical characteristics contribute to severe infections. In the other 2 sets of analysis, we predicted severity and mortality of COVID-19 at the population level, considering individuals not known to be infected as “controls.” Our objectives are twofold. The first is to build prediction models for severity and mortality from COVID-19. In addition, we will uncover how different risk factors and their interactions impact on disease severity.

## Methods

### UK Biobank Data

The UKBB is a large-scale prospective cohort comprising nearly 500,000 individuals aged 40-69 when they were recruited in 2006-2010. Given that the first case of COVID-19 in the UK was recorded on January 31, 2020, individuals with recorded mortality before January 31, 2020 (28,931 out of 502,524 individuals) were excluded. We also excluded from subsequent analyses a very small number of individuals (n=19) whose cause of mortality was COVID-19 (ICD code U07.1) but with negative test result(s) within 1 week. The current age of individuals included in our analyses ranged from 50 to 87 years, with 50.77% (255,170/502,524) being older than 70. This analysis was conducted under the project number 28732. For details of the UKBB data, please also refer to Sudlow et al [19].

### COVID-19 Phenotypes

COVID-19 outcome data were downloaded from data portal provided by the UKBB. Details of data release are provided in [20]. Briefly, the latest COVID test results were extracted on December 30, 2020 (last update on December 14, 2020). The data set also included an indicator on whether the patient was an inpatient when the specimen was taken. We consider inpatient (hospitalization) status as a proxy for severity, as more sophisticated indicators of severity cannot be reliably derived yet. We noted that only 10.22% (468,235/4,581,006 infected cases, from [21] as of June 16, 2021) of patients were admitted in the UK; as such, it is likely that only the more severe cases were hospitalized. Hospitalization has also been considered as an outcome measure in many studies, including those of vaccination effectiveness [22-25], risk prediction [26,27], and genetic/clinical risk factors [28,29] underlying severe COVID-19.

In general, we required both test result and origin to be 1 (indicating positive test and inpatient origin, respectively) to qualify as an “inpatient” case. For a small number of individuals with inpatient origin=0 and result=1, but changed to origin=1 with result=0 within 2 weeks’ time (based on the fact that median duration of viral persistence is nearly 2 weeks [30]), we still considered those as inpatient cases (ie, assume the hospitalization was related to the infection). All other patients with at least one positive SARS-CoV-2 test result were considered as “outpatient.”

Data on mortality and cause of mortality were also extracted (with latest update on December 14, 2020). Individuals with recorded cause of mortality as “U07.1” were considered as having a fatal infection with laboratory-confirmed COVID-19 (please also refer to [31]). We defined a case as “severe COVID-19” if the individual is an inpatient or if the cause of mortality is U07.1.

### Sets of Analysis

Four sets of analysis were performed. The first 2 sets were restricted to test-positive cases (n=7846). “Severe COVID-19” (n=2386) and death (n=477) due to COVID-19 were treated as outcomes. Because only prediagnostic clinical data were available, the main objective of this analysis was to identify baseline risk factors for severe/fatal illness among the infected. We then performed another 2 sets of analysis with the same outcomes, but the “unaffected” group was composed of the general population (n=465,728) that did not have a diagnosis of COVID-19 or were tested negative. The 4 sets of analysis were also referred to as cohorts A-D as shown in Table 1. We also constructed gender-specific prediction models.

**Table 1:** The four sets of analysis performed and predictive performances (full model and lite model).

Cohort	Group 1	Group 2	n (group 1)	n (group 2)	Area under the curve^a^ (%)		95% CI (%)	
					Full	Lite	Full	Lite
A	Hospitalized or fatal cases	Nonhospitalized cases	2386	5460	72.3	71.6	71.1-73.6	70.3-72.9
B	Fatal cases	All other COVID-19 cases	477	7369	81.4	81.8	79.1-83.8	79.4-84.2
C	Hospitalized or fatal cases	UK Biobank patients without a COVID-19 diagnosis or tested negative	2386	465,728	69.6	69.6	68.4-70.8	68.4-70.7
D	Fatal cases	UK Biobank patients without a COVID-19 diagnosis or tested negative	477	465,728	82.5	83.0	80.2-84.8	80.8-85.3

^a^AUC was taken from the average of 5 folds of cross-validation.

### Variables Included in Analysis

We extracted a total of 97 clinical variables of potential relevance based on the literature. For details, please refer to Table S1d in Multimedia Appendix 1 and the references therein. The prediction model using all 97 variables will be referred to as the “full” model, as opposed to a simplified model (“lite” model; see below) based on mainly demographic data and medical history that can be more readily obtained. Among the 97 variables, 21 were categorical and 76 were quantitative traits. The missing rates of variables were all below 20% (ranging from 0.0% to 19.9% for the 97 variables). We included a wide range of clinical features here, with an objective to uncover potential novel risk factors for the disease. The ML model we employed (XGboost) tends to have a low bias and high variance; however, with proper tuning of hyperparameters and regularization, overfitting can be largely avoided even when a large number of predictors are included [32].

The full list of variables is shown in Table S1b in Multimedia Appendix 1. Briefly, we included basic demographic variables (eg, age, sex, ethnic group, socioeconomic status as indicated by the Townsend deprivation index [TDI]), comorbidities (eg, heart diseases, type 1 and 2 diabetes mellitus [T1DM/T2DM], hypertension [HT], asthma/chronic obstructive pulmonary disease [COPD], cancer, dementia, and psychiatric disorders), indicators of general health (number of medications taken [cnt_tx], number of illnesses, etc.), blood measurements (hematology, liver and renal function measures, metabolic parameters such as lipid levels, HbA1c), anthropometric measures (eg, waist circumference [WC], waist-to-hip ratio [WHR], body mass index), and lifestyle risk factors (eg, smoking, drinking habits). Disease traits were defined based on ICD-10 diagnoses (UKBB data-field 41270), self-reported illnesses (UKBB data-field 20002), and data from follow-ups. Individuals with no records of the relevant disease from either self-reports or ICD-10 diagnoses were regarded as having no history of the disease.

### Imputation

Missing values of remaining features were imputed with the R package missRanger (R Foundation). The program is based on missForest [33], which is an iterative imputation approach based on random forest. It has been widely used and has been shown to produce low imputation errors and good performance in predictive models [34]. The main difference between missRanger and missForest is that the former uses the R package “ranger” to build random forests, which can lead to a large improvement in speed. Predictive mean matching (pmm) was also employed to avoid imputation with values not present in the original data. We employed the default parameters (pmm.k=3, num.trees=100) and default settings of ranger. Out-of-bag errors (in terms of classification errors or normalized root-mean-squared error) were computed which provides a guide to imputation accuracy.

We have also attempted to use multiple imputation by chained equation (MICE) for imputation. For our data set with nearly 500,000 individuals, MICE stopped after running for 6 hours due to memory overflow error (>64 GB), whereas missRanger finished the imputation within 3 hours successfully. We considered the computational burden of MICE as too high and therefore employed missRanger in our analyses.

Several studies have compared MissForest with MICE, and there are several advantages of missForest. For categorical variables, imputation accuracy of missForest is likely to be higher than that of MICE [35]. MissForest also runs considerably faster than MICE and is especially suitable for imputation settings where complex interactions and nonlinear relationships are likely [33]. Stekhoven et al [33] reported superior performance of missForest compared with MICE, with reduction in the proportion of falsely classified entries of up to 60%. In another comparison study, missForest and MICE performed similarly but it was reported that highly correlated variables may lead to significant problems with MICE [36].

### XGboost Prediction Model

XGboost with gradient-boosted trees was employed for building prediction models. Analysis was performed by the R package “xgboost.” We employed a fivefold nested cross-validation strategy to develop and test the model. To avoid overoptimistic results due to choosing the best set of hyperparameters based on test performance, the test sets were *not* involved in hyperparameter tuning.

In each iteration, we divided the data into 5 folds, among which one-fifth was reserved for testing only. For the remaining four-fifth of the data, we further sampled four-fifth for training and one-fifth for hyperparameter tuning. The best prediction model was applied to the test set. The process was repeated 5 times. A grid-search procedure was used to search for the best combination of hyperparameters (eg, tree depth, learning rate, regularization parameters for L1/L2 penalty). The full range of hyperparameters chosen for grid search is given in Table S6 in Multimedia Appendix 1.

### Building a Simplified “Lite” Model

The “full” model described above covers a wide range of predictors but some features (such as blood biochemistries) may not be readily accessible. For easier implementation in practice, we also built a simplified prediction model (also referred to as the “lite” model) based on a reduced set of 27 predictors. The reduced set of variables were chosen based on the ease of being assessed or measured, which included comorbidities (see above), anthropometric measures (BMI, weight, WC), demographic variables (eg, age, sex, ethnic group), and general indicators of health (number of medications taken, number of illnesses).

### Evaluating Predictive Performance and Calibration

To evaluate the predictive performance of the prediction models, we computed the area under the receiving-operating characteristic curve (AUC–ROC), which is very widely used in clinical prediction studies. We also calculated other measures including the area under the precision–recall curve (AUC–PRC), F1 score, accuracy, and Matthews correlation coefficient (MCC). The cutoff of predicted probability for calculating the latter 3 measures was determined by optimizing the geometric mean of sensitivity and specificity.

In addition to good ability to discriminate cases from noncases, it is also important that the predicted event probabilities match with the observed probabilities (also known as calibration of a model). We assessed calibration by several measures, including the Hosmer–Lemeshow test, expected calibration error (ECE), and maximum calibration error (MCE) [37-40] across 10 equally sized bins by discretizing the predicted probabilities. We also attempted 3 approaches to further improve calibration, including Platt scaling, isotonic regression, and beta calibration [41-44]. The objective is to rescale the predicted probabilities such that they are closer to the actual probabilities of the outcome [45].

### Identifying and Quantifying the Effects of Important Predictors

In this work we primarily employed Shapley value (ShapVal) [46,47] to assess variable importance, which is a measure based on game theory to assess the contribution of each feature. ShapVal has been shown to represent a consistent and locally accurate contribution of each feature [48]. ShapVal enables local explanation of the model as it could be computed for each observation, but can also provide global importance measures. By contrast, gain and split count may produce inconsistent estimates of global importance as shown by Lundberg et al [48].

Intuitively, the ShapVal of the *i*th feature (for individual *k*) is the contribution of this feature to the prediction of outcome for the individual, averaging over all possible orderings of the features (as the contribution may differ when variables enter the prediction algorithm in different orders). We ranked the global importance of features based on mean absolute ShapVal as described in previous studies [46,47]. We also attempted an alternative approach similar to “permutation importance” proposed in [49]. This method involves permuting the outcome vector to model the distribution of ShapVal under the null, and comparing the null ShapVals with the observed ShapVal. We derived a *P* value from permutation as an alternative indicator of feature importance. A total of 500 permutations were performed for each model. To verify the validity of the permutation procedure especially under imbalanced case–control data, we also carried out a small-scale simulation study. A data set with 50,000 individuals and 10 covariates (*x*_1_, *x*_2_, ..., *x*_10_) were generated, where the first covariate *x*_1_ was linearly correlated with the outcome*.* The control-to-case ratio was set at 976:1, same as that for cohort D. Type I error and power were assessed by repeating the entire permutation procedure for 100 randomly generated data sets (please see Multimedia Appendix 2 for details).

A related index is the Shapley *interaction* value [47], which computes the difference in Shapley value of feature *i* with and without another feature *j*. ShapVal were averaged across 5 folds. Besides, we included the “gain” measure for reference, which is the reduction of loss or impurity contributed by all splits by a specific variable.

An advantage of Shapley value is that it is calculated for each individual, so how each risk factor affects a specific person’s risk of infection/severity can be estimated as well. To illustrate this concept, we also produced decision plots for individuals at the highest, median, and lowest risk of each cohort.

### Cluster Analysis Based on Shapley Value

We also performed cluster analysis based on ShapVal to identify subgroup of patients who share similar clinical risk factors with respect to severity of infection. As introduced in [48], this approach may be considered a form of “supervised” clustering, as the outcome (severe/fatal disease) is also taken into account in the clustering process. Unlike a traditional clustering approach based on risk factors, this approach has important advantages. First, the clusters derived may be more clinically relevant as the *outcome* is also considered, reducing the chance that irrelevant features contribute to the subgrouping (an irrelevant feature will have relatively small variations in ShapVal and will not contribute substantially to clustering). Second, this approach essentially considers all features on the same “scale,” as ShapVal is computed with respect to the outcome. Input features are often of different units and scales, but ShapVal considers feature contributions to the outcome as the unit of measure. Because of computational cost concerns, here we only performed clustering on cohorts A (nonsevere vs severe infection) and B (fatal vs nonfatal infection).

### K-Means Sparse Clustering

Here we performed k-means sparse clustering to uncover underlying patient subgroups based on ShapVal of risk factors. As the number of features included is large but not all may contribute to the underlying subgroups, we employed *sparse* clustering which incorporates feature selection in the clustering process. The R package “sparcl” was employed. To perform sparse k-means clustering, we need to predetermine the number of clusters and tuning parameter (L1 penalty) for feature selection [50]. The optimal number of clusters was assumed to be the same as that in k-means clustering, which was determined by the silhouette index. The tuning parameter (L1 bound) was set to range between 2 and 6 with an interval of 0.4. Then the gap statistic [51] was used to determine the optimal tuning parameter.

## Results

An overview of the sample sizes in each set of analysis is presented in Table 1. Please also refer to Table S1a and S1b in Multimedia Appendix 1 for a detailed summary of case counts and covariates.

### Simulation Results for the Permutation Testing Approach

Simulation results for the validity of permutation *P* values are presented in Table S8 in Multimedia Appendix 1. We observed no inflation of type I error (false-positive rate) despite the imbalanced case-to-control ratio. At a *P* value threshold of 0.05, the proportion of results with *P*<.05 for *x*_2_ to *x*_10_ (variables with null effect) remained less than 0.05 for different effect sizes of the predictor (please also refer to Multimedia Appendix 2).

### Prediction Performance of the XGboost Model for Risk and Severity of Infection

#### AUC–ROC and Other Results

We performed 5-fold cross-validation and the average AUC under the ROC curve is given in Table 1 and Table S2a in Multimedia Appendix 1. Here we describe the results for the full models first. We observed better predictive performances in cohorts B (fatal cases vs outpatient cases) and D (fatal cases vs population with no known infection), where fatalities from COVID-19 were modeled. The corresponding mean AUC–ROC values were 0.814 (95% CI 0.791-0.838) and 0.825 (95% CI 0.802-0.848), respectively. The mean AUC–ROC for cohort A (hospitalized/fatal cases vs other cases) was 0.723 (95% CI 0.711-0.736) and that for cohort C (hospitalized/fatal cases vs population with no known infection) was 0.696 (95% CI 0.684-0.708).

As for the “lite” models which included a reduced set of predictors, the predictive performances in terms of AUC are broadly similar, with estimated AUC–ROC for cohorts A-D of 0.716, 0.818, 0.696, and 0.830, respectively.

The results of other predictive indices are listed in Table S2b in Multimedia Appendix 1. Estimates of AUC–PRC were the highest for cohorts A and B (0.535 and 0.171, respectively) and much lower for cohorts C and D (0.007 and 0.006, respectively). This is expected due to the much higher prevalence of outcome in the first 2 cohorts. AUC–PRC may be approximated by the average precision (please refer to [52] for further details).

We also conducted sex-stratified analysis (Table S2a in Multimedia Appendix 1). The resulting AUC–ROC was similar to the overall analysis in males (except for cohort D), but generally lower in females. This may be partially explained by lower number of severe and fatal cases in females, which leads to greater difficulty in model training.

#### Proportion of Cases Explained by Individuals at the Top k% of Predicted Risk

We also computed the proportion of cases explained by individuals at the highest *k*% of predicted risks (Table 2). For example, considering the full model, for prediction of mortality among infected individuals (cohort B), individuals at the highest 5%, 10%, and 20% of predicted risks explain 17.4% (83/477), 32.7% (156/477), and 52.0% (248/477) of total fatalities, respectively. As for prediction in the population (cohort D), individuals at the highest 5%, 10%, and 20% of predicted risks explain 32.5% (155/477), 45.7% (218/477), and 63.5% (303/477) of total fatalities, respectively. For prediction of severe disease among the infected (cohort A), individuals at the highest 5%, 10%, and 20% of predicted risks explain 11.2% (267/2386), 21.6% (515/2386), and 38.2% (911/2386) of total cases, respectively, while more than half (1272/2386, 53.3%) of cases are explained by people at the top 30% of predicted risks. For prediction of severe cases in the population (cohort C), the corresponding figures were 19.7% (470/2386), 29.3% (700/2386), and 42.7% (1019/2386), respectively, and more than half (1260/2386, 52.8%) of cases are explained by people at the top 30% of predicted risks. Similar figures were observed for full and lite models in general.

These results showed in general a strong enrichment of cases among those predicted to have high risks, indicating good model discriminatory ability.

**Table 2:** Relative risk (RR) comparing subjects in the top and bottom k% of predicted risks and proportion of cases explained by those at top k% of predicted risk.

Full model							Lite model			
*k*		Risk in top k%^a,b^	Risk in bottom k%	RR	Proportion of cases explained by top k%	Risk in top k%^a,b^		Risk in bottom k%	RR	Proportion of cases explained by top k%
**Cohort A**										
	5	0.676	0.148	4.56	0.112	0.691		0.158	4.37	0.113
	10	0.654	0.138	4.74	0.216	0.644		0.157	4.10	0.211
	20	0.579	0.145	4.00	0.382	0.581		0.153	3.79	0.381
	30	0.540	0.148	3.65	0.533	0.533		0.152	3.50	0.526
	40	0.489	0.152	3.20	0.644	0.479		0.158	3.03	0.630
	50	0.443	0.166	2.67	0.730	0.439		0.170	2.59	0.720
**Cohort B**										
	5	0.214	0.000	Infinity	0.174	0.212		0.003	84.27	0.174
	10	0.200	0.001	158.20	0.327	0.216		0.008	28.38	0.352
	20	0.171	0.008	22.42	0.562	0.188		0.008	24.59	0.618
	30	0.148	0.009	16.57	0.727	0.155		0.008	19.21	0.763
	40	0.127	0.009	14.21	0.830	0.131		0.009	14.23	0.866
	50	0.111	0.010	10.94	0.916	0.111		0.011	10.37	0.912
**Cohort C**										
	5	0.0201	0.0017	11.76	0.197	0.0210		0.0013	15.88	0.207
	10	0.0149	0.0021	6.98	0.293	0.0158		0.0012	12.95	0.310
	20	0.0109	0.0023	4.67	0.427	0.0118		0.0021	5.71	0.462
	30	0.0090	0.0030	2.99	0.528	0.0097		0.0027	3.57	0.573
	40	0.0075	0.0033	2.27	0.590	0.0084		0.0026	3.20	0.656
	50	0.0069	0.0033	2.09	0.678	0.0074		0.0028	2.63	0.725
**Cohort D**										
	5	0.0067	0.00000	Infinity	0.325	0.0068		0.00000	Infinity	0.333
	10	0.0047	0.00002	218.02	0.457	0.0047		0.00006	73.67	0.463
	20	0.0033	0.00011	30.30	0.635	0.0032		0.00009	36.75	0.616
	30	0.0026	0.00014	18.74	0.746	0.0027		0.00011	23.38	0.784
	40	0.0021	0.00016	13.17	0.828	0.0022		0.00013	16.68	0.874
	50	0.0018	0.00022	8.35	0.893	0.0019		0.00015	13.03	0.929

^a^‘Top k%’ refers to top k% of *predicted* probability of outcome by XGboost.

^b^‘Risk in top k%’ refers to the actual probability of the outcome (severe disease or fatality) within the patients belonging to the highest k% of predicted risks.

#### Relative Risk of Actual Outcome Probabilities, Comparing Those at the Highest and Lowest k% of Predicted Risks

We also computed the relative risk (RR) of infection or severe disease by comparing individuals at the highest and lowest *k*% of predicted risks (Table 2). For example, considering the full model, if we compare the actual probability of outcome at the top decile (top 10%) against those at the bottom decile of predicted risks, the RR was 4.74, 158.2, 6.98, and 218.02, respectively, for cohorts A to D. If we compare the top 20% against the lowest 20% of predicted risks, the corresponding RRs were 4.00, 22.42, 4.67, and 30.30. The RRs for the lite model were similar for cohorts A and C, but were smaller for cohorts B and D when the comparison was made at the more extreme ends of predicted risks.

We observed large RRs for cohorts B and D, suggesting that the prediction models were able to discriminate individuals at the highest and lowest risks of fatality very well. RRs for cohorts B and D were much larger than those for cohorts A and C, indicating that the model predicted fatality better than severe disease.

#### Calibration

As for calibration, please refer to Figures S6 and S7 in Multimedia Appendix 3. For full models, cohort A was well-calibrated (without using other methods for recalibration) with ECE of 0.022 and MCE of 0.044 only. For other models, the ECE and MCE were generally larger, probably due to large difficulty in calibration with a much lower probability of the outcome. The best ECEs (after recalibration by 1 of the 3 methods) were 0.11, 0.14, and 0.02, respectively, for cohorts B-D. The Hosmer–Lemeshow test was nonsignificant in cohorts C and D (*P*=.99 and .98, respectively). For the “lite” models, the best ECEs were 0.017, 0.043, 0.024, and 0.089, respectively, for cohorts A-D, with nonsignificant Hosmer–Lemeshow test results except for cohort B (Hosmer–Lemeshow *P*=.49, .003, .97, and .41 for cohorts A-D, respectively).

### Results From Cluster Analysis Based on ShapVal

**Figure 1:**
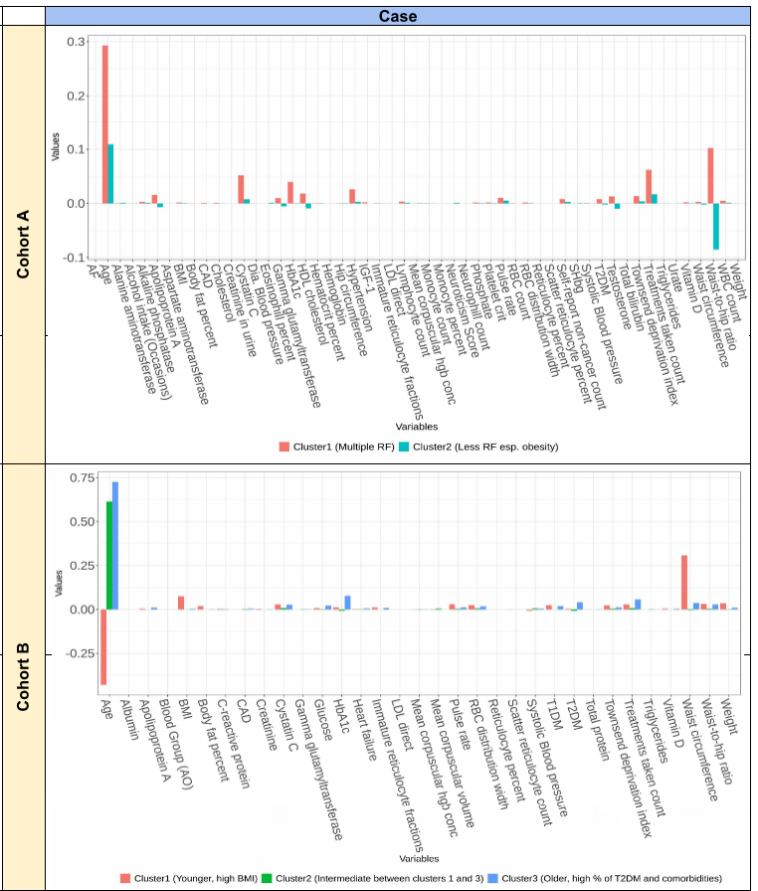
Results of sparse k-means clustering based on Shapley values (ShapVal) in cohorts A (hospitalized cases) and B (fatal cases). The y-axis indicates the ShapVal and only those risk Factors with significant differences (*P*<.05 in t-test or ANOVA) across clusters were shown on the x-axis. AF: atrial fibrillation; CAD: coronary artery disease; Hb: hemoglobin; HbA1c: hemoglobin A1c; HDL: high-density lipoprotein; LDL: low-density lipoprotein; RBC: red blood cell; RF: risk factor; SHBG: sex hormone binding globulin; T1DM: type 1 diabetes; T2DM: type 2 diabetes.

Figures 1 and S11 in Multimedia Appendix 3 show the results based on sparse k-means clustering. We performed clustering separately in cases and controls to uncover patient subgroups with different clinical background. Here we focus on clustering results within cases. As the number of variables is large, we only showed the variables that were statistically significant (*P*<.05 from *t* test or ANOVA) across the clusters in the figures. For cohort A, we found 2 clusters as the optimal solution. The first cluster has higher ShapVal for most risk factors, especially age, but also cnt_tx, HbA1c, cystatin C, high-density lipoprotein (HDL)-cholesterol, and HT. ShapVal for WHR was positive for the first group but negative for the second group. The first cluster may represent a subgroup of severe cases with a larger number of clinical risk factors/comorbidities and advanced age, while the second cluster may be a distinct group with less conventional risk factors (especially obesity), yet is susceptible to severe infections perhaps due to other (unmeasured) factors, such as genetics.

Considering cohort B cases (fatal infections), the optimal solution comprised 3 clusters. Interestingly, the first and third clusters seemed to be markedly different with respect to their risk factor profiles. Mean ShapVal for age was largely negative for the first cluster but highly positive for the other 2 clusters. By contrast, mean ShapVal for WC was markedly higher and positive for the first cluster. The third cluster was characterized by the highest mean ShapVal for age, and higher (positive) ShapVal for mainly cnt_tx, HbA1c, and T2DM. The results suggest that there may exist pathophysiologically distinct subgroups of patients with fatal infection. The first cluster represents a subgroup with younger age but with higher proportion of obesity. The third cluster represents another subgroup with advanced age, more comorbidities, and higher proportion of glucose abnormalities or T2DM. The second cluster is in between.

### Important Contributing Variables Identified

#### Overview

**Figure 2:**
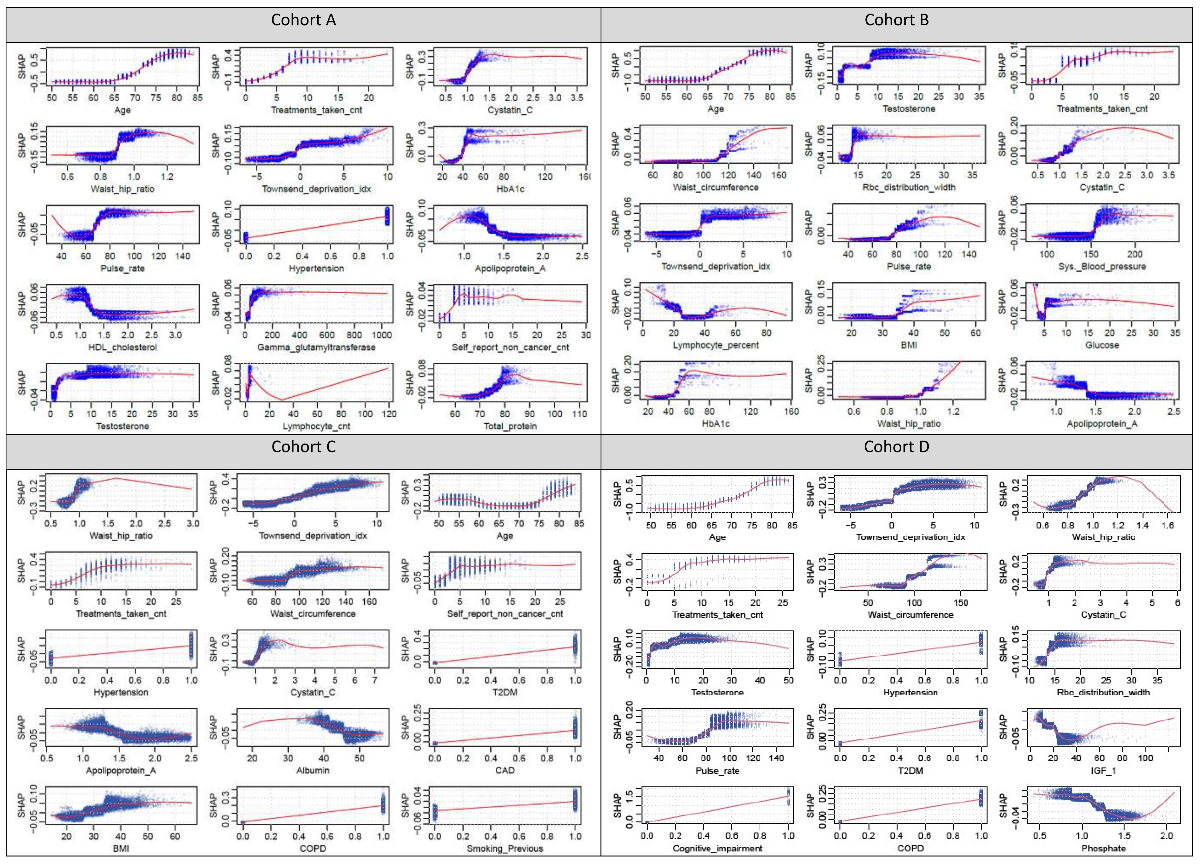
Shapley value dependence plots of the top 15 risk factors ranked by mean abs(shapley value) (full model) for cohorts A, B, C, and D, respectively. Shapley value (y-axis) is computed on a log-odds scale. Every unit increase of ShapVal corresponds to an odds ratio (OR) of exp(1)=2.72 compared with the baseline. Positive ShapVal indicates increase in the odds of the outcome and vice versa. CAD: coronary artery disease; COPD: chronic obstructive pulmonary disease; HDL: high-density lipoprotein; RBC: red blood cell; T2DM: type 2 diabetes mellitus.

**Figure 3:**
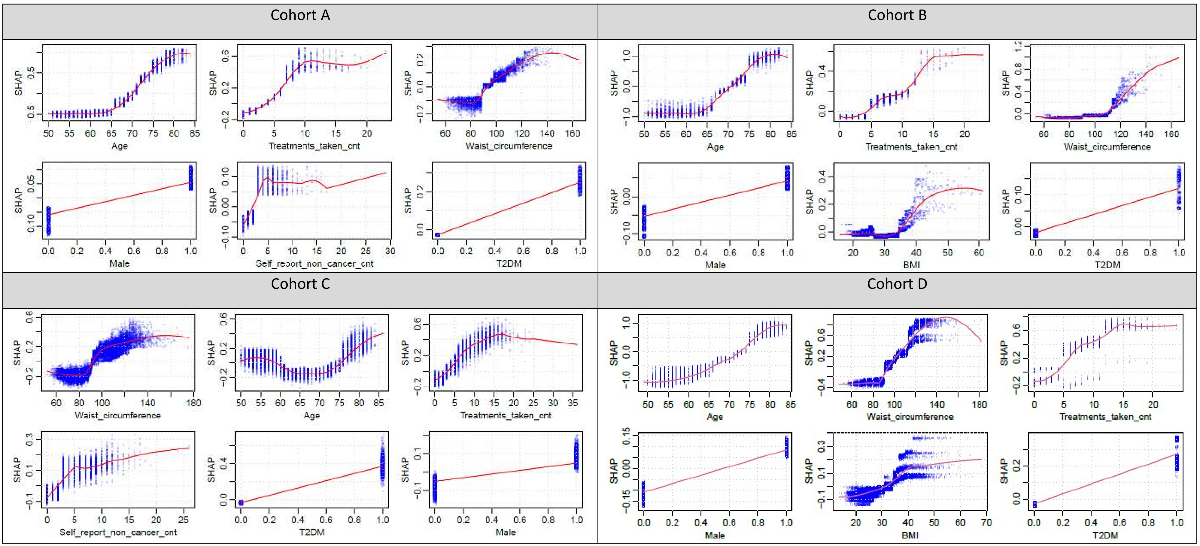
ShapVal dependence plots of the top 6 risk factors ranked by mean abs(shapley value) (lite model) for cohorts A, B, C, and D, respectively. T2DM: type 2 diabetes mellitus.

Here we primarily report the results of the full model as a more complete set of predictors is included. The Shapley dependence plots (ranked by mean absolute ShapVal) of the top 15 features (full model) are shown in Figure 2 and those of the top 6 features for the lite model are presented in Figure 3. For more complete plots (up to 30 variables) with ranking by mean abs(ShapVal) or permutation *P* values, please refer to Figures S1-S4 in Multimedia Appendix 3.

Full ShapVal analysis results on all variables are given in Tables S3a-c in Multimedia Appendix 1. The top 10 variables (ranked by either ShapVal or permutation *P* value) from the full model are presented in Tables 3 and 4 while the top 5 from the lite model are presented in Tables 5 and 6. We also included variable importance by gain, and plots are presented in Figure S5a and S5b in Multimedia Appendix 3.

**Table 3:** Top 10 risk factors ranked by mean absolute Shapley value for cohorts A, B, C, and D (full model).

Risk factor			ShapVal		*P* value
**Cohort A**					
	Age	0.442		.002	
	Treatments taken count	0.093		.002	
	Cystatin C	0.088		.002	
	Waist-to-hip ratio	0.085		.002	
	Townsend deprivation index	0.059		.004	
	HbA1c^a^	0.056		.002	
	Pulse rate	0.048		.002	
	Hypertension	0.048		.002	
	Apolipoprotein A	0.027		.016	
	HDL^b^ cholesterol	0.026		.016	
**Cohort B**					
	Age	0.708		.002	
	Testosterone	0.069		.002	
	Treatments taken count	0.048		.002	
	Waist circumference	0.035		.002	
	RBC^c^ distribution width	0.027		.002	
	Cystatin C	0.024		.002	
	Townsend deprivation index	0.023		.002	
	Pulse rate	0.019		.004	
	Systolic blood pressure	0.016		.002	
	Lymphocyte percentage	0.015		.004	
**Cohort C**					
	Waist-to-hip ratio	0.113		.002	
	Townsend deprivation index	0.096		.002	
	Age	0.088		.002	
	Treatments taken count	0.063		.002	
	Waist circumference	0.044		.002	
	Self-report: noncancer count	0.043		.002	
	Hypertension	0.036		.002	
	Cystatin C	0.030		.024	
	T2DM	0.030		.002	
	Apolipoprotein A	0.024		.052	
**Cohort D**					
	Age	0.519		.002	
	Townsend deprivation index	0.136		.002	
	Waist-to-hip ratio	0.131		.002	
	Treatments taken count	0.115		.002	
	Waist circumference	0.110		.002	
	Cystatin C	0.096		.002	
	Testosterone	0.086		.002	
	Hypertension	0.061		.002	
	RBC distribution width	0.046		.002	
	Pulse rate	0.036		.006	

^a^HbA1c: hemoglobin A1c.

^b^HDL: high-density lipoprotein.

^c^RBC: red blood cell.

^d^T2DM: type 2 diabetes mellitus.

**Table 4:** Top 10 risk factors ranked by P-value, listing only factors which are not yet included in for cohorts A, B, C, and D (full model).

Risk factor		*P* value	ShapVal	
**Cohort A**				
	T2DM^a^	.004	0.010	
	Self-report: noncancer	.008	0.018	
	Depression	.008	0.004	
	CAD^b^	.016	0.002	
	Cancer diagnosed by doctor	.026	0.000	
	Alcohol intake (occasions)	.028	0.002	
	AF^c^	.028	0.000	
	Smoking (current)	.036	0.000	
	γ-glutamyltransferase	.046	0.021	
	WBC^d^ count	.046	0.014	
**Cohort B**				
	BMI	.002	0.015	
	Glucose	.002	0.015	
	HbA1c^e^	.002	0.014	
	Weight	.002	0.010	
	Mean platelet volume	.002	0.009	
	T2DM	.002	0.007	
	Sleep duration	.002	0.006	
	T1DM^f^	.002	0.003	
	Cognitive impairment	.002	0.003	
	CAD	.002	0.003	
**Cohort C**				
	COPD^g^	.002	0.015	
	Depression	.002	0.009	
	Cognitive impairment	.002	0.007	
	CAD	.004	0.017	
	Ethnic (Asian/Asian British)	.004	0.007	
	Heart failure	.004	0.007	
	AF	.004	0.006	
	Smoking (previous)	.006	0.015	
	Stroke	.012	0.001	
	Ethnic (Black/Black British)	.020	0.001	
**Cohort D**				
	T2DM	.002	0.026	
	Cognitive impairment	.002	0.024	
	COPD	.002	0.021	
	AF	.002	0.016	
	Heart failure	.002	0.007	
	CAD	.002	0.008	
	Ethnic (Black/Black British)	.004	0.004	
	Stroke	.004	0.002	
	Alcohol drinker (current)	.004	0.001	
	Smoking (previous)	.006	0.003	

^a^T2DM: type 2 diabetes mellitus.

^b^CAD: coronary artery disease.

^c^AF: atrial fibrillation.

^d^WBC: white blood cell.

^e^HbA1c: hemoglobin A1c.

^f^T1DM: type 1 diabetes mellitus.

^g^COPD: chronic obstructive pulmonary disease.

**Table 5:** Top 5 risk factors ranked by mean absolute Shapley value for cohorts A, B, C, and D (lite model).

Risk factor		ShapVal	*P* value
**Cohort A**			
	Age	0.496	.002
	Treatments taken count	0.121	.002
	Waist circumference	0.085	.002
	Male	0.058	.002
	Self-report: noncancer count	0.054	.004
**Cohort B**			
	Age	0.721	.002
	Treatments taken count	0.079	.014
	Waist circumference	0.071	.040
	Male	0.048	.010
	BMI	0.034	.242
**Cohort C**			
	Waist circumference	0.153	.002
	Age	0.120	.002
	Treatments taken count	0.102	.002
	Self-report: noncancer count	0.064	.002
	T2DM^a^	0.050	.002
**Cohort D**			
	Age	0.056	.002
	Waist circumference	0.248	.002
	Treatments taken count	0.154	.002
	Male	0.098	.002
	BMI	0.043	.036

^a^T2DM: type 2 diabetes mellitus.

**Table 6:** Top 5 risk factors ranked by *P* value, listing only factors which are not yet included in for cohorts A, B, C, and D (lite model).

Risk factor			*P* value	ShapVal
**Cohort A**				
	T2DM^a^	.002		0.047
	Smoking (current)	.004		0.026
	Depression	.016		0.015
	Alcohol drinker (current)	.020		0.013
	CAD^b^	.022		0.010
**Cohort B**				
	T2DM	.006		0.027
	Cognitive impairment	.006		0.015
	T1DM^c^	.020		0.009
	Bipolar	.024		0.006
	AF^d^	.036		0.011
**Cohort C**				
	COPD^e^	.002		0.024
	Ethnic (Asian/British Asian)	.002		0.016
	Cognitive impairment	.002		0.008
	Male	.004		0.049
	CAD	.004		0.023
**Cohort D**				
	T2DM	.002		0.043
	COPD	.002		0.039
	Cognitive impairment	.002		0.029
	AF	.002		0.024
	Ethnic (Black/Black British)	.002		0.016

^a^T2DM: type 2 diabetes mellitus.

^b^CAD: coronary artery disease.

^c^T1DM: type 1 diabetes mellitus.

^d^AF: atrial fibrillation.

^e^COPD: chronic obstructive pulmonary disease.

**Figure 4:**
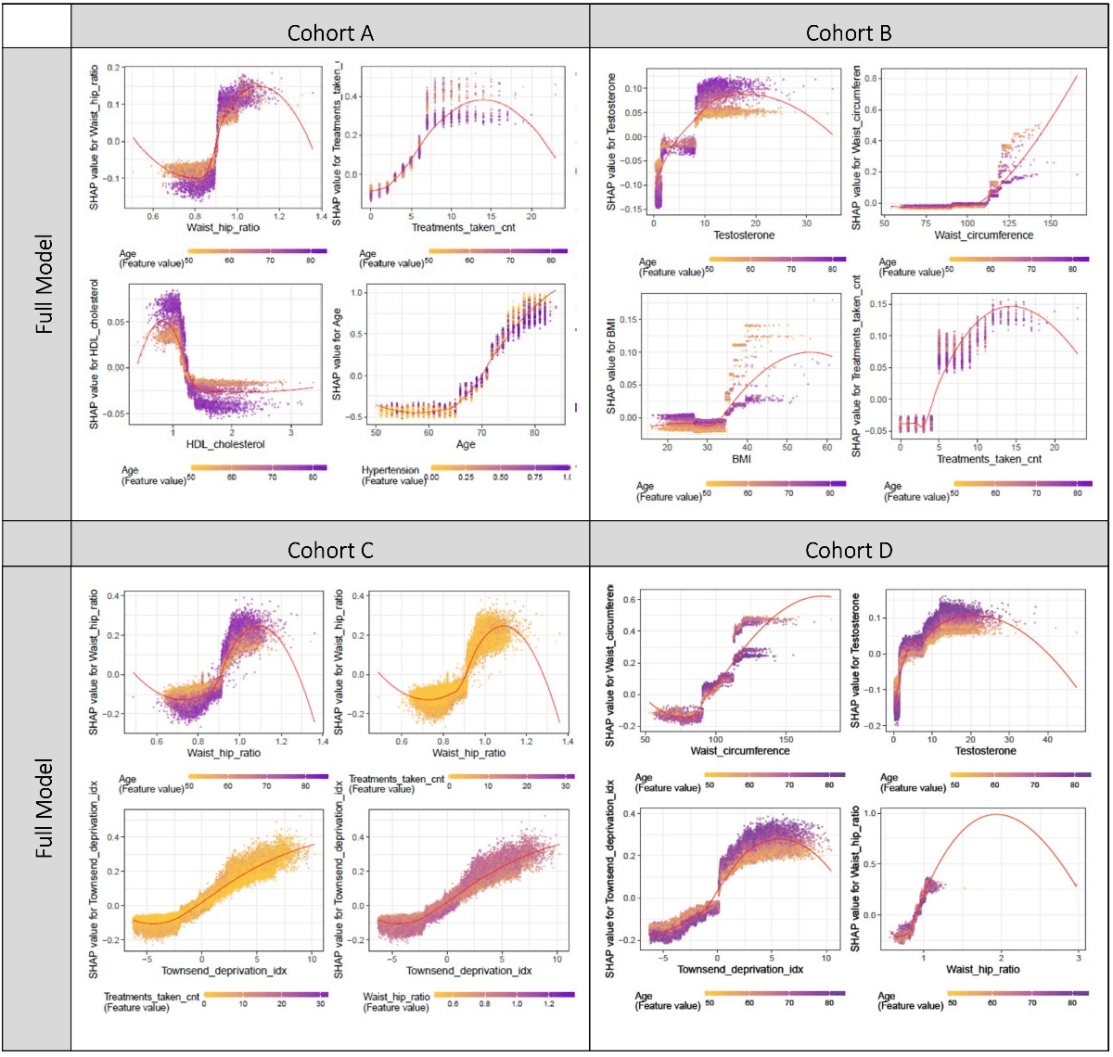
ShapVal interaction plots of the full model for the top 4 interacting pairs of cohorts A, B, C, and D, respectively.

As for interaction analyses, top results are presented in Table 7 and full results in Tables S4 and S5 in Multimedia Appendix 1. Plots are presented in Figure 4 (top 2 interacting pairs from each model) and Figures S8 and S9 in Multimedia Appendix 3 (top 6 interacting pairs).

Note that ShapVal is measured on the log-odds scale. Every unit increase of ShapVal corresponds to an odds ratio of exp(1)=2.72. Positive ShapVal indicates increase in the odds of outcome and vice versa.

**Table 7:** Top interacting pairs of variables ranked by ShapVal (full model).

Risk factor 1		Risk factor 2	Value
**Cohort A**			
	Waist-to-hip ratio	Age	150
	Treatments taken count	Age	149
	HDL^a^ cholesterol	Age	86
	Age	Hypertension	85
	Cystatin C	Age	84
**Cohort B**			
	Testosterone	Age	195
	Waist circumference	Age	95
	BMI	Age	82
	Treatments taken count	Age	63
	Pulse rate	Age	57
**Cohort C**			
	Waist-to-hip ratio	Age	709
	Waist-to-hip ratio	Treatments taken count	494
	Townsend deprivation index	Treatments taken count	481
	Townsend deprivation index	Waist-to-hip ratio	450
	Albumin	Waist-to-hip ratio	407
**Cohort D**			
	Waist circumference	Age	859
	Testosterone	Age	780
	Townsend deprivation index	Age	725
	Waist-to-hip ratio	Age	603
	Age	Hypertension	585

^a^HDL: high-density lipoprotein.

#### Cohort A (Hospitalized/Fatal Cases vs Outpatient Cases)

The top 5 contributing features by ShapVal included age, number of medications received (cnt_tx), cystatin C, TDI, and WHR, followed by HbA1c. Higher levels of these risk factors generally lead to higher disease severity among the infected. Interestingly, Shapley dependence plots revealed potential *nonlinear* and “threshold” effects of risk factors on the outcome. For example, age of 65 or above was associated with a markedly increased risk of severe/fatal infection. Markedly elevated risks were also observed for HbA1c levels over 40 mmol/mol and 5 or more drugs received. Impaired renal function (IRF; raised cystatin C >1 mg/L) was also linked to worse outcomes. For WHR, levels of 0.9 or higher appeared to be associated with a marked increase in risks. For other features, please refer to Figure 2. We note that at the extreme ends of variables, the observations are often sparse, so the trend shown by the Loess curve may not be reliable (this also applies to other cohorts). Variable importance based on gain revealed similar patterns of important features (Figures S5a and S5b in Multimedia Appendix 3).

If we consider the “*P* value” or permutation importance (PermImp) measure, variables with top 10 (absolute) ShapVal also showed significant *P* values (*P*<.05 for all cases). T2DM was among the top 10 by PermImp but not by ShapVal. Depression and coronary artery disease (CAD) also showed low *P* values (*P*<.02), but were not listed among the top 30 by ShapVal.

Regarding interactions between variables, most of the top interacting pairs involved age (Figure 4 and Tables S4 and S5 in Multimedia Appendix 1). For example, younger individuals were observed to have more extreme ShapVal at similar ranges of cnt_tx. The effect of WHR on severity was more marked among the elderly, and the same was true for HDL-cholesterol (low HDL is a risk factor).

#### Model B (Fatal Cases vs Outpatient Cases)

The top 5 contributing variables by ShapVal included age, testosterone (which may reflect the effect of gender), cnt_tx, WC, and red blood cell distribution width (RDW), which were followed by cystatin C, TDI, pulse rate, systolic blood pressure (SBP), and percentage of lymphocytes. Again, certain nonlinear and “threshold” effects appeared to be present for many top-ranked features. For age, the risk for mortality was more marked beyond 65 years. Higher levels of all the above risk factors (RFs) (except percentage of lymphocytes, which showed a U-shaped relationship) were associated with higher mortality, but the effects were nonlinear. Regarding the top results based on PermImp, 8 out of 10 predictors ranked high by ShapVal also had the lowest *P* values (lowest *P* value of .002 since we performed 500 permutations). Other top-ranked features (*P*=.002) included HbA1c, type 1 and T2DM, weight, mean platelet volume, etc.

Variable importance based on gain yielded similar results (Figures S5a and S5b in Multimedia Appendix 3). As for interactions between the variables, again interactions were most prominent with age (Figure 4). For example, the effects of WC and BMI (when exceeding a threshold of around 110 cm and 35 kg/m^2^, respectively) on mortality were more prominent among younger individuals. The effects of testosterone and HbA1c, however, were more marked in older individuals.

#### Model C (Hospitalized/Fatal Cases vs Population With No Known Infection)

Based on ShapVal, WHR was the top contributing variable and WC was ranked fifth, suggesting that central obesity may be a stronger predictor for severe disease than BMI alone (BMI was ranked 13th by ShapVal). As before, TDI and age were ranked among the top. For age, slightly unexpectedly, a U-shaped curve was observed, which suggests lowest risk at the age group of 65-70. Note that model C may also capture RFs related to susceptibility to infection. It is possible, for instance, that younger individuals had higher risks of exposure due to work or social interactions. Among the top 10, two are related to general multiple comorbidities (cnt_tx and cnt_noncancer). Increased cystatin C and lower apolipoprotein A were also associated with higher susceptibility to severe infections, and HT and T2DM were also among the top 10. Considering PermImp as the ranking criteria, COPD, depression, and dementia were observed to have the lowest permutation *P* values (*P*=.002) though not top listed by ShapVal.

The interaction plot (Figure 4) shows WHR may interact with age, with elderly individuals showing more prominent effects from changes in WHR.

#### Model D (Fatal Cases vs Population With No Known Infection)

Based on ShapVal, age was the top feature, followed by TDI, WHR, number of drugs taken, and WC. Other top features included cystatin C, testosterone, HT, RDW, and pulse rate. Higher levels of these features (or presence of comorbidity) generally lead to higher mortality risks. Based on PermImp, T2DM, dementia, and COPD were the most highly ranked (ignoring features that are already listed in the top 10 by ShapVal).

Shapley interaction analysis suggested that the top interacting pairs involved age and some of the top contributing features (Figure 4 and Figure S8 in Multimedia Appendix 3). The effects of testosterone (likely also reflects gender effects) and TDI were more prominent among the elderly, while the effect of BMI was larger in the younger age groups.

As for important variables from the sex-stratified analysis, the top variables were similar which included age, WC/WHR, cystatin C, number of medications received, socioeconomic status (as reflected by TDI), among others (Table S3c in Multimedia Appendix 1).

#### PermImp Compared With ShapVal

Overall speaking, the PermImp measure tends to rank binary traits higher than ShapVal. Of note, several diseases were consistently top listed by PermImp across the 4 cohorts (though some were not highlighted by ShapVal), including CAD, atrial fibrillation (AF), T2DM, and dementia, which were among the top 10 in at least three cohorts in Table 4. Other diseases that were listed at least twice included depression, COPD, stroke, and heart failure.

### Results From the “Lite” Model

Here we highlight top contributing features for the “lite” models consisting of 27 predictors (Table S3b in Multimedia Appendix 1). Remarkably, the top 3 features (ranked by ShapVal) were consistent across all 4 cohorts. These features included age, cnt_tx, and WC (WHR was not included in the lite model as WC is easier to measure). Of note, sex and T2DM were ranked among the top 6 across all cohorts.

If we consider PermImp as the ranking criteria (further ranked by ShapVal if PermImp is equal), age, cnt_tx, and WC were still highly ranked and listed among the top 5 in at least three cohorts (Table S3b in Multimedia Appendix 1). T2DM was ranked among the top 5 in all cohorts. Other potential risk factors included dementia (top 10 across 3 cohorts) as well as AF, COPD, and CAD (top 10 across 2 cohorts).

### Results From the Logistic Model

As discussed above, we primarily focused on the XGboost ML model as it can capture nonlinear relationships and interactions between predictors. Here we also performed our analyses with logistic regression (LR) for comparison. For prediction performance (Table S7a in Multimedia Appendix 1), the AUC–ROC of the full LR model was 0.728 (95% CI 0.715-0.741), 0.810 (95% CI 0.786-0.834), 0.712 (95% CI 0.701-0.724), and 0.833 (95% CI 0.810-0.856), respectively, for cohorts A-D. For the “lite” model (using 27 predictors only), the AUC–ROC of the LR approach was 0.722 (95% CI 0.709-0.735), 0.824 (95% CI 0.801-0.848), 0.697 (95% CI 0.685-0.709), and 0.834 (95% CI 0.812-0.857), respectively, for cohorts A-D (Table S7a in Multimedia Appendix 1). These figures were very close to those obtained by XGboost, although AUC–ROC using LR was slightly higher in general (median difference=0.005). If we compute the RR of individuals at the highest and lowest k% of predicted risks, the results were generally similar (Table S7b in Multimedia Appendix 1). For cohort D and the full model of cohort B, XGboost performed better than LR at the extreme ends of predicted risks, with observed risk=0 (ie, no cases were observed) for those predicted at the lowest 5% of risk (Table 2).

While prediction is one of our goals, uncovering important contributing factors and their relationship to COVID-19 severity is a major objective of this study. In fact, the latter is considered our primary objective when considering the analyses within patients infected (cohorts A and B). As LR assumes linearity on a log-odds scale, it could not capture nonlinear relationships or “threshold effects” of variables on disease severity.

### Individual Shapley Decision Plots and Online Calculator

We also showed individual Shapley decision plot for 3 individuals with the highest, median, and lowest predicted risks in each cohort (Figure S10 in Multimedia Appendix 3). The y-axis is based on a log-odds scale.

To facilitate further research and studies on risk-prediction models, we also constructed an online risk calculation tool (for “lite” model) [53]. The online tool can also construct a Shapley decision plot based on individual risk factors.

## Discussion

### Principal Findings

In this study we have performed 4 sets of analysis, predicting severe or fatal COVID-19 infection among affected individuals or in the population. We observed good predictive power from the XGboost ML models, especially for the prediction of mortality. We also identified risk factors for increased severity or mortality, and uncovered possible nonlinear effects of some features, which may be clinically relevant and shed light on disease mechanisms.

### Prediction of Severity/Mortality

In general, our prediction models achieved reasonably good predictive power. The models predicted mortality (AUC 81%-83%) better than severity of disease. As discussed earlier, in the absence of better alternatives, hospitalization (test performed as inpatient) was used as a proxy for severity. However, reasons or criteria for hospitalization may vary across individuals or hospitals, and some tests may be performed in inpatients for surveillance or due to other confirmed/suspected cases in the ward. As a result, hospitalized patients could also include some with mild or moderate illnesses, which may also impair the prediction performance. By contrast, mortality from infection is a more objective outcome. Other studies (eg, [54-56]) have also defined “severe” or “critical” disease based on intensive care unit admission or need for ventilatory support. However, we could not find sufficiently detailed clinical data to support such a classification at the time of this analysis.

### Discriminatory Power of the Models and Clinical Implications

By assessing the proportion of cases explained by those at the top k% of predicted risks, we observed in general a strong enrichment of cases among those with high predicted risks, indicating good discriminative ability of the models and suggesting the possibility to focus on the highest-risk group for targeted preventions or treatment. A similar strong enrichment was also observed for the lite model with fewer predictors. We also observed large RRs of the actual outcomes when comparing individuals at high and low percentiles of predicted risks. For example, for the prediction of mortality among the infected, the RR was up to 158 times (~20% vs 0.1%) when comparing and top and bottom deciles using the full model, and 28.38 times when considering the simplified model (~21% vs 0.8%). These results suggest that the prediction models may be used for risk stratification and prioritizing those at higher risks of deterioration, for early medical attention or admission. As the “lite” model only relies on demographic data and information on comorbidities, risk stratification may be conducted even at the start of the illness without other blood or imaging results.

### Previous Relevant Works

A number of studies have focused on prediction of severity/mortality of COVID-19 (corresponding to our prediction in cohorts A and B) and were reviewed in [8]. For cohort A (prediction of severity among infected), the AUC is 72.3%, which is moderate but not as good as many previous ML models for severity prediction [8]. The AUC for prediction of mortality is much higher (AUC=81.4%), although we noted that some studies have reported higher predictive power from clinical symptoms, blood biochemistry on admission, and imaging features [8]. We understand that without access to the above features, predictive performance may be inferior. By contrast, due to heterogeneity of clinical samples, treatment approaches, model evaluation methods, and other features across studies, direct comparisons of predictive performance across studies may be difficult. Here we are not aiming at deriving a highly accurate prediction model; the main purpose is to identify general or “baseline” risk factors for severe disease, thereby gaining insight into disease pathophysiology. However, we also showed that such clinical features or blood measurements, even when collected much earlier in time, may still be highly predictive of outcomes and hence may be incorporated into existing prediction algorithms. The models here may also be useful when blood results or imaging are not available (eg, before admission) and the goal is to quickly classify a patient’s risk.

For cohorts C and D, the general population (with no known infection) was treated as “controls.” Compared with cohorts A and B, the identified risk factors may also increase the overall susceptibility to infection. The AUC for cohort C (severe/fatal disease) is about 70% but is much higher when mortality is considered as the outcome (AUC=~83%). To our knowledge, there are still very few predictive models built at a *general* *population* *level* to identify susceptible individuals; this work is among the first to employ an ML approach to predict the risk of COVID-19/severe infection at a population level. DeCaprio et al [57] proposed an ML model to assess the vulnerability to COVID-19 in the population. However, due to limited data, no actual COVID-19 patients were included and “proxy” outcomes were used instead. Models were built from mainly demographic and comorbidity data to predict hospitalization due to acute respiratory distress syndrome, pneumonia, influenza, acute bronchitis, and other respiratory tract infections.

Another very recent study (“QCOVID” study) from the UK [58] utilized general practice records from 6.08 million adults (age 19 to 100) as the derivation cohort and 2.17 million adults as the validation set. Mortality from COVID-19 was the primary outcome and a survival model (subdistribution hazard model) [58] was used to predict mortalities. The predictors included demographic (eg, age, TDI, ethnicity), lifestyle (eg, BMI, smoking), and a large range of comorbid conditions. The resulting Harrell’s C (comparable to AUC) was 0.928. However, we note that the QCOVID study included individuals of a much younger age range (19 or older), which will improve predictive performance, as age is by far the most important predictor of mortality, with markedly reduced risks in younger individuals. For example, if we refer to age-specific predictive performance (see Supplementary Table C in the study [58]), Harrell’s C for mortality was 0.678, 0.831, 0.812, and 0.814 in 50-59, 60-69, 70-79, and 80+ year olds, respectively, for males in the first follow-up period (January 24 to April 30, 2020). For females, the corresponding numbers were 0.618, 0.77, 0.866, and 0.821. These numbers reflect lower predictive power when restricted to a narrower age range. One main difference between this work and the above study is that we employed an *XGboost ML* approach which is able to also capture nonlinear and more complex interaction effects. As shown in our Shapley dependence plots, the models were able to reveal nonlinear effects in a data-driven manner. We also included a number of blood measurements to shed light on potential new risk factors and mechanisms underlying the disease. The QCOVID study employed a survival model (subdistribution hazard) that accounts for time-to-event and competing risks; however, the proportional hazards assumption is required which may not hold due to restrictions/interventions introduced during the period (ie, time-dependent associations may be present).

A few other studies have investigated risk factors (especially comorbidities) for COVID-19 infection in the UKBB. For example, Atkins et al [12] studied elderly individuals (age >65) in UKBB, and found that HT, history of falls, CAD, T2DM, and asthma were the top comorbidities among hospitalized cases. The analysis was restricted to the elderly population, however. In a more recent work, McQueenie et al [13] studied the impact of multiple comorbidities and polypharmacy on infection risks. Having 2 or more long-term conditions, cardiometabolic disorders, and polypharmacy were associated with heightened risks of infection. Among individuals with multiple comorbidities, severe obesity and IRF may lead to increased risks. Another study of primary care patients in the UK revealed that deprivation, male sex, older age, ethnicity (being Black), and chronic renal disease were associated with higher risks of being tested positive [59]. Another large-scale British primary care study of more than 17 million individuals revealed similar risk factors as above [60]. There is also a relatively large literature on the study of risk factors associated with severe or fatal disease [15-18,61-64]. Some commonly reported risk factors included age, sex, obesity, T2DM, HT, renal, cardiometabolic, and respiratory disorders. As discussed above, an important difference between the above epidemiological studies and this work is that we employed XGboost, an ML approach that can uncover nonlinear and interaction effects, while other studies mostly employed regression models that assume linear and additive effects of covariates. We also performed a comprehensive analysis including 4 models covering different outcomes and both infected and population cohorts.

### Comparison With Logistic Model

We have performed LR to compare with XGboost on cohorts A and B. The differences in predictive performance appeared to be small. The number of cases (especially fatalities) is relatively small in this data set, and this may limit the predictive performance of more complex models such as XGboost, which may be expected to improve with larger case numbers. An important advantage of XGboost is that it can detect nonlinear relationships when compared with LR. In addition, XGboost may handle multiple collinearity better than LR. Assuming 2 highly correlated features A and B, for each specific tree usually only 1 variable will be used and as the trees are sequential, the focus of the model will be usually on one but not on both features [65]. Hence, XGboost also handles multicollinearity well, which is important here as many clinical variables are correlated. XGboost also directly models interaction between variables. It is much more difficult for LR to model interactions due to the rapid increase in feature space when interaction terms are included.

### Highlights of Potential Risk Factors

For the limit of space, we shall only highlight the top 5-10 risk factors ranked by ShapVal here. Across the 4 cohorts, age and cardiometabolic risk factors predominate the top risk factors. Age and WHR/WC were ranked among the top 5 across all 4 cohorts. The number of medications taken was among the top 5 across all cohorts, and cystatin C (reflecting renal function) was among the top 10 across all cohorts. HbA1c was a top 10 risk factor for cohort A, and T2DM was also highly ranked across multiple cohorts especially when PermImp was considered. TDI (reflecting socioeconomic status) was among the top 10 in most cohorts. As described above, results from the “lite” models were generally in line with those from the full models, with age, WC, and cnt_tx consistently ranked as the top 3.

Obesity has been observed to be a major risk factor for susceptibility or severity of infection in the UKBB [14,66] and in many other studies [67,68]. The observation that WC/WHR were highly ranked suggests that *central* obesity is a major risk factor and may be a better predictor of severity than BMI alone.

Another major risk factor we identified is IRF, as reflected by elevated risks with raised urea and cystatin C. Several studies also suggested that IRF increases risk of mortality [64,69,70], although it is probably not as widely recognized as cardiometabolic disorders as a major risk factor. Because COVID-19 itself may lead to renal failure, our findings specifically suggest that underlying or baseline IRF is an important risk factor. The high ranking of cystatin C also indicates that this measure may better reflect renal function than urea or creatinine (which were also included in our analysis) [71,72], and may serve as a superior predictor for COVID-19 severity.

Other potential risk factors briefly highlighted below were less reported. As some were listed only once or twice among the top 10, and for some their ShapVal was close to other risk factors, further replications are required. For example, testosterone was top ranked by XGboost (for mortality), with higher levels associated with increased risk. This may partially reflect that males are at a higher risk of fatal infections, but it remains to be studied whether testosterone itself is involved in the pathophysiology of severe COVID-19, as the ML model chose this variable instead of sex. Studies have suggested that elevated or reduced testosterone levels may be associated with a more severe clinical course [73]. Besides, interestingly, 5-alpha-reductase inhibitors or androgen-deprivation therapy has been shown to be associated with a lower risk or severity of disease [74,75]. We also found a few hematological indices that may be potential risk factors. High RDW was associated with mortality in our study and was also identified in a recent meta-analysis of 3 studies as a risk factor [76]. Low lymphocyte percentage was a top 10 risk factor in cohort B, which may be related to immune functioning and response to infections. Lymphopenia was reported as a main hematological finding in those with severe illnesses [40,77]. Most previous studies considered hematological indices at admission or during hospitalization. Slightly surprisingly, this study suggested that high RDW or reduced lymphocyte percentage *prior to the diagnosis* may also be predictive of worse outcomes.

### Comorbid Diseases Associated With Severity as Highlighted by PermImp

Among the diseases being included as covariates, T2DM is most consistently ranked among the top, no matter whether full or lite models are used, and regardless of ranking by ShapVal or PermImp (*P* value). T2DM has been shown in numerous studies to be associated with higher risk and severity of infection [78,79]. We noted some discrepancy between the ranked results based on ShapVal and those based on PermImp. In general, the latter measure favors binary variable, while ShapVal alone tends to rank continuous variables higher. We are unsure about the exact reason, but it may be an interesting topic for further methodology studies. If we employed a composite ranking criteria based on PermImp followed by ShapVal, then a few more diseases were ranked among the top 10, such as hypertension and COPD. For cohort D, T2DM, dementia, COPD, AF, heart failure, and CAD were also top ranked, suggesting that a range of chronic cardiovascular, respiratory, and neuropsychiatric conditions may be associated with increased mortality.

### Full and Lite Prediction Models

We note that the simplified (lite) prediction model has very similar predictive performance (as assessed by AUC) to the “full” model with a larger panel of predictors. However, it is important to note that features associated with the outcome may not always improve predictive power. AUC is relatively insensitive to detecting changes in predictive performance when additional risk factors are added [80-82].

For example, Pencina et al [80] showed that in the prediction of cardiovascular disease risk in a study on women’s health, adding extra established risk factors often result in minimal improvements in AUC. For instance, in a model with age, SBP, and smoking, adding any lipid measures result in only an increase of 0.01 in AUC from the baseline of 0.76. In the same vein, starting from a full prediction model [containing Ln(age), Ln(SBP), smoking, Ln(Total cholesterol), Ln(HDL)], deleting any one of these established risk factors (except age) resulted in a very small reduction in AUC of <0.02. In general, for a model with high baseline AUC from existing predictors (eg, age, sex, and obesity in the case of COVID-19), including additional predictors may not result in much improvement in discriminative power or AUC [83].

Nevertheless, it is still valuable to study variable importance (eg, ShapVal) from the ML model as it may shed light on the pathophysiology of the disease*.* For example, many factors such as age and T2DM may lead to poorer renal function (and higher cystatin C), which in turn may increase the severity of infection. Given that age, T2DM, and other main comorbidities are already modeled, adding cystatin C may not improve discriminative power of the model. However, its inclusion may still change the predicted probability of outcome, which will be reflected in ShapVal. The high ranking of cystatin C (based on ShapVal) may shed light on renal impairment as a potential mechanism associated with clinical deterioration.

Some limitations have been discussed above; for example, the use of hospitalization as a proxy for severity, and that the predictors were recorded prior to the pandemic. We briefly discuss other limitations here. The UKBB is a very large-scale study with detailed phenotypic data, but still the number of fatal cases is relatively small. In addition, the UKBB is not entirely representative of the UK population, as participants tend to be healthier and wealthier overall [84]. Further, it remains to be studied whether the findings are generalizable to other populations. Symptom measures and lung imaging features were not available at the time of analysis. Despite adjusting for a rich set of predictors and that all predictors were recorded prior to the outbreak, causality cannot be confirmed from this study, due to risk of residual confounding by unknown factors. This study was performed on a cohort with age over 50, and generalizability to younger individuals remains to be studied. In cohorts C and D, the population with no known infection was regarded as controls. It is expected that some may become infected in the future, and some may have been infected but not tested; however, the chance of missing cases of severe infection is probably not high. Since the UKBB represents a relatively healthy population with a low rate of severe COVID-19 cases so far (236/468,114, 0.50%), we expect the use of “unscreened” controls is unlikely to result in substantial bias.

Regarding the ML model, XGboost is a state-of-the-art method that has been consistently shown to be the best or one of the best ML methods in supervised learning tasks/competitions [85] (especially for tasks not involving computer vision or natural language processing). Nevertheless, other ML methods may still be useful or may uncover novel risk factors. Assessing variable importance is a long-standing problem in ML; here we mainly employed ShapVal, which is both computationally fast and was shown to have good theoretical properties [46,47].

### Conclusions

In conclusion, we identified a number of baseline risk factors for severe/fatal infection by an ML approach. Shapley dependence plots revealed possible nonlinear and “threshold” effects of risk factors on the risks of infection or severity. To summarize, age, central obesity, IRF, multiple comorbidities, cardiometabolic abnormalities or disorders (especially T2DM), and low socioeconomic status may predispose to poorer outcomes, among other risk factors. The prediction models (of cohorts C/D) may be useful at a population level to identify those susceptible to developing severe/fatal infections, thereby facilitating targeted prevention strategies. Further replication and validation in independent cohorts are required to confirm our findings.

## Appendix

Multimedia Appendix 1 Supplementary tables S1-S8.

Multimedia Appendix 2 Simulation experiment to verify the validity of permutation testing for rare outcomes.

Multimedia Appendix 3 Supplementary figures.

## Abbreviations

AF: atrial fibrillation
AUC–PRC: area under the precision–recall curve
AUC–ROC: area under the receiving-operating characteristic curve
CAD: coronary artery disease
COPD: chronic obstructive pulmonary disease
ECE: expected calibration error
HDL: high-density lipoprotein
HT: hypertension
IRF: impaired renal function
LR: logistic regression
MCC: Matthews correlation coefficient
MCE: maximum calibration error
MICE: multiple imputation by chained equation
ML: machine learning
RDW: red blood cell distribution width
RF: risk factor
RR: relative risk
SBP: systolic blood pressure
T1DM: type 1 diabetes mellitus
T2DM: type 2 diabetes mellitus
TDI: Townsend deprivation index
UKBB: UK Biobank
WC: waist circumference
WHR: waist-to-hip ratio
