# Supplementary material for: Uncovering Clinical Risk Factors and Predicting Severe COVID-19 Cases Using UK Biobank Data: Machine Learning Approach
Source: JMIR Public Health Surveill. 2021 Sep 30;7(9):e29544. doi: 10.2196/29544 (PMC8485986; doi:10.2196/29544)
Supplement: Multimedia Appendix 2 [file publichealth_v7i9e29544_app2.docx]

**Supplementary Text**

**Simulation experiment to verify the validity of permutation testing for rare outcomes**

To verify that the permutation procedure gives a valid estimate of the significance of predictors even when the outcome is highly imbalanced, we carried out a small simulation study. A dataset with 50,000 subjects and 10 covariates (*x*_1_, *x*_2_…*x*_10_) was filled with values generated by the rnorm() function in R, where the first covariate *x*_1_ was linearly correlated with the outcome*.* The rest of the variables (*x*_2_ *to x*_10_) have no association with the outcome. The outcome is modeled by *Y* = *b*_0_ + *b*_1_*x*_1_ + *b*_2_*x_2_ +…b*_10_*x*_10_ + *e,* where *e* follows a normal distribution with mean=0 and variance=2. A cutoff threshold was chosen so that the control-to-case ratio in the simulated dataset was equal to 976:1, the same control:case ratio as in cohort D.

An XGboost model was built from this dataset by the same method as described in the section “XGboost prediction model”. The ShapVal of the 50,000 subjects were evaluated as usual. Five hundred permutations were performed to obtain each permutation p-value. The entire permutation testing procedure was repeated for 100 randomly generated datasets, and the proportion of results with permutation p-value<0.05 for each covariate was evaluated (Table S8).

We observed no inflation of type I error (false positive rate) despite the imbalanced case:contol ratio. At a p-value threshold of 0.05, the proportion of results with p<0.05 for *x*_2_ *to x*_10_ remained less than 0.05 for different values of *b*_1_. In other words, the type I error is maintained properly. The power to detect *x*_1_ as a significant predictor ranged from 0.91 to 1.
